# Novel clinical device tracking and tissue event characterization using proximally placed audio signal acquisition and processing

**DOI:** 10.1038/s41598-018-30641-0

**Published:** 2018-08-13

**Authors:** Alfredo Illanes, Axel Boese, Iván Maldonado, Ali Pashazadeh, Anna Schaufler, Nassir Navab, Michael Friebe

**Affiliations:** 1 Otto-von-Guericke-Universität, INKA Intelligente Katheter, Magdeburg, Germany; 2 Technische Universität München, Fakultät für Informatik, München, Germany

## Abstract

We propose a new and complementary approach to image guidance for monitoring medical interventional devices (MID) with human tissue interaction and surgery augmentation by acquiring acoustic emission data from the proximal end of the MID outside the patient to extract dynamical characteristics of the interaction between the distal tip and the tissue touched or penetrated by the MID. We conducted phantom based experiments (*n* = 955) to show dynamic tool/tissue interaction during tissue needle passage (a) and vessel perforation caused by guide wire artery perforation (b). We use time-varying auto-regressive (TV-AR) modelling to characterize the dynamic changes and time-varying maximal energy pole (TV-MEP) to compute subsequent analysis of MID/tissue interaction characterization patterns. Qualitative and quantitative analysis showed that the TV-AR spectrum and the TV-MEP indicated the time instants of the needle path through different phantom objects (a) and clearly showed a perforation versus other generated artefacts (b). We demonstrated that audio signals acquired from the proximal part of an MID could provide valuable additional information to surgeons during minimally invasive procedures.

## Introduction

Medical interventional devices (MIDs) such as needles, catheters or guide wires are frequently used to provide access for minimally invasive therapy and diagnosis^1^. These procedures include applications such as tissue biopsies, brachytherapy seed placement, regional anaesthesia, vascular catheter-based procedures, and percutaneous tumour therapies like radiofrequency or cryo ablation^1,2^.

Generally, in all these procedures the operator needs to introduce the MID into the body and guide it toward the target site following a prediagnostically determined or intraoperatively image guided path without unwanted damaging of important structures or organs. Percutaneous needle insertion damaging healthy tissue or blood vessels and guide wire vessel perforation in vascular procedures are typical examples of that. The deeper the target site is and the closer it is to sensitive structures, the more complicated the procedure becomes. The clinicians skill and experience is an essential pre-requirement, but even combined with real time and live external (e.g. Ultrasound, X-ray) or internal (Optical) guidance, imaging still comes with error potential due to a multitude of imaging artefacts. For example the biopsy of an unintended tissue region can result in a false negative diagnosis, or in case of brachytherapy seed placement, the radiation dose would be wrongly applied and the tumour not properly irradiated. Additional problems come with the use of preoperative imaging data due to changed patient position and patient motion.

The accuracy of the procedure could be improved by adding data related to the interaction forces that are developed between a MID and the perforated tissue. For example, when a needle is inserted into soft tissue, interaction forces are developed at the needle tip and along the needle shaft when the MID passes through different tissue layers such as skin, muscle, and fat. Several sensor based approaches have been proposed for MID/tissue interaction assessment for haptic feedback in needle and guide wires. Most of them are based on measuring force using piezoresistive sensors^1–6^. Other works proposed piezoelectric and optical sensors for measuring pressure^7,8^, impedance^9,10^ and piezoelectric transducers to measure blood flow velocity for monitoring guide wire vessel occlusion^11,12^. The main drawback of these approaches is that the sensors are usually located at the distal end of the guide wire, i.e., invasively in the part of the device that is inserted inside the body. Moreover, due to this distal placement characteristic they come with a degradation of the devices clinical efficiency due to the size of the sensors and placement of required cables or wires.

In this work we propose a new approach for MID/tissue interaction monitoring and surgery augmentation using acoustic emission (AE) data acquisition from the proximal end of a conventional clinically used device to extract dynamical characteristics of the interaction between the distal tip and the tissue. The hypothesis is that a change of tissue that occurs when the MID passes through with the distal end causes changes in the characteristics of the measured signal at the proximal end. By applying advanced signal processing techniques to the acquired audio signal it should be possible to identify and characterize significant events related to the interaction of the MID distal tip with the tissue, such as penetration, friction and puncture dynamics. In contrast with recent papers on surgical soundtracks or auditory display^13,14^, which use medical image analysis for sonification, this work aims at acquiring, modifying and amplifying natural sounds of tool/tissue interactions.

AE techniques have been extensively used as a non-invasive diagnosis tool in different research and development fields. In mechanical machining processes AE has been widely used for non-invasive online tool condition monitoring^15^, where AE signals are processed for tool wear assessment or in general for machine health monitoring and fault diagnosis. Other areas such as nondestructive testing use AE techniques for detecting and locating flaws in structures^16^. In medicine, with the exception of the areas of orthopedy^17^, AE has been little exploited. Some few works have been proposed with a goal similar to our approach^18–20^, but only for acquiring AE from drilling machines in orthopaedic surgery or in general for bone surgery. Our approach is intended to be used for soft tissue applications and involves a completely different challenge.

Experimental setups for two different MIDs, a biopsy needle and a guide wire were implemented and a time-varying autoregressive (TV-AR) modelling has been used for extracting valuable features from the audio signal related to the friction dynamics between the tool and the tissue. Some preliminary results of this work concerning the guide wire experimental setup has been presented in^21^.

Qualitative and quantitative results showed that with the proposed approach the time instants when the needle enters and leaves different phantom objects are clearly identifiable and also showed signal patterns that are significantly different between perforation and generated artefacts for the guide wire. This demonstrates that audio signals by itself acquired from the proximal part of a conventional and only little altered MID could potentially provide valuable additional information to surgeons during minimally invasive procedures. In combination with established imaging guidance this could lead to increased success of selected surgical procedures or reduce false negative events. This could also be used in a future setup for readjusting robotic devices that exclusively rely on preoperative imaging data.

## Methods

### Experimental setup and AE data acquisition

Experimental setups (ES) for two different MIDs, a 18G 200 mm length biopsy needle with the needle core (ITP, Germany) and a 0.014−*inch* guide wire of 1.8 *m* length (Boston Scientific, US) placed inside a flushed 1.9 *F* micro catheter of 1.5 *m* length were implemented. The main objective of the ES for the biopsy needle was to observe audio signal dynamical changes when the needle passes through two different tissue structures. In contrast the guide wire ES intended to analyse signal dynamics of perforation in vascular structures.

**Figure 1:**
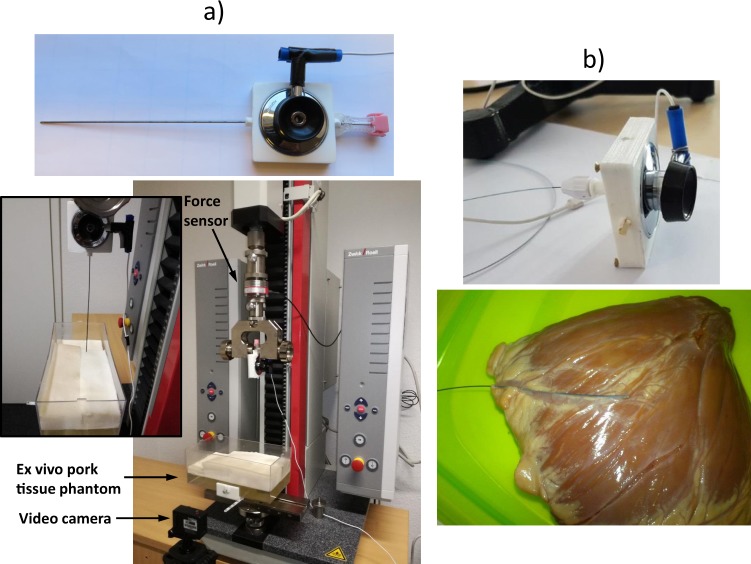
(**a**) Connection between the stethoscope and the needle and automatic needle insertion in a gelatine phantom filled with *ex-vivo* porcine tissue. (**b**) Guide wire connected to the stethoscope and insertion in a porcine heart vessel (adapted from^21^).

For both experiments AE signals were acquired using a stethoscope connected to a microphone which was directly and firmly attached to the proximal end of the MID via a 3D printed adapter (see top of Fig. 1). For each MID experiment qualitative and quantitative analysis were performed and a database for each ES was implemented for the quantitative case.

A gelatine phantom filled with different fruits, chicken breast and liver located 6 cm deep was used for the biopsy needle qualitative analysis, while for quantitative analysis the gelatine phantom was filled with *ex-vivo* porcine tissue. The needle insertion was performed manually for qualitative analysis and automatically for quantitative analysis.

The qualitative and quantitative guide wire perforation tests were performed on *ex-vivo* porcine coronary arteries. The guide wire was placed inside a flushed micro catheter. This was shaped to mimic a natural tortuous pathway to the coronaries.

For both ES, the audio signals were recorded in WAV format with a sampling frequency of 44100 Hz. The *Matlab R2015b* was used for the audio signal analysis.

#### Database implementation for needle experimental setup quantitative analysis

For the needle ES, 80 audio recordings were acquired during automatic insertion of the biopsy needle into *ex-vivo* porcine tissue phantom using a testing machine (Zwicki, Zwick GmbH & Co.KG, Ulm) at an insertion velocity of 3 mm/s (see Fig. 1a).

**Figure 2:**
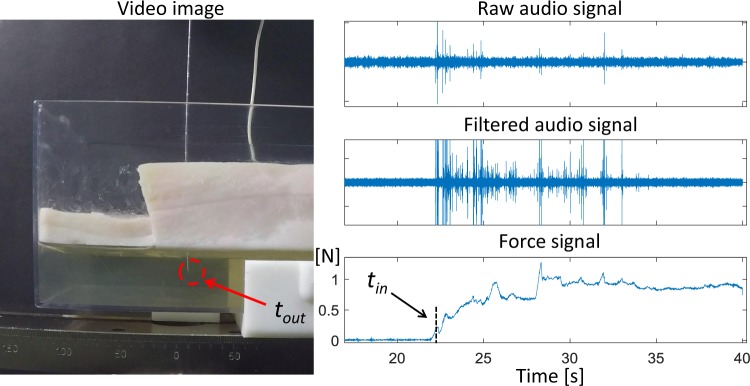
Determination of time instants of tissue entry and exit and synchronization of the video with the audio.

In order to show repeatability of the approach the time instants of object entry *t*_*in*_ and exit *t*_*out*_ were manually annotated. For that the force from the testing machine was recorded synchronous with the audio and a video camera was placed in front of the phantom (see Fig. 1a). The main objective of that was to set *t*_*in*_ at the time instant of first signal deflection when the force started to change (contact of the needle with the tissue) and to synchronize this time instant with the one observed when the needle touches the tissue in the video. In this way video and audio were also synchronized and *t*_*out*_ was taken directly from the synchronized video (see Fig. 2).

We have performed experiments at two additional velocities in order to study the performances of our approach when the needle insertion velocity is changed. The insertion velocities encountered during current clinical procedures can have large variation but in^2^ insertion velocities between 1 and 10 mm/s were analysed. Experiments at 5 mm/s and 8 mm/s were performed additionally to the set of 3 mm/s described above. We have acquired 20 recordings for each additional velocity and the same procedure than the experiments at 3 mm/s was applied.

#### Database implementation for guide wire experimental setup quantitative analysis

For the guide wire ES, 560 audio signals of 30 seconds duration were recorded during the tip perforation of coronary arteries belonging to 10 porcine hearts (see Fig. 1b). The main objective of the created database was to analyse performances on classifying the audio signals as a perforation or as an artefact. Therefore, 315 additional recordings with different types of induced guide wire audio artefacts were performed, including friction between the guide wire and the artery wall (165 recordings) and tiny guide wire bumps (150 recordings).

### Audio signal characteristics extraction

**Figure 3:**
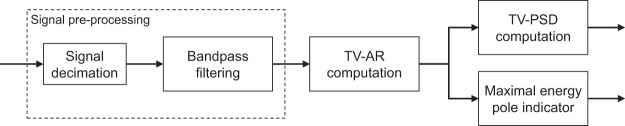
Main steps of the signal processing algorithm for AE signal analysis.

The block diagram of Fig. 3 displays the main steps of the used signal processing approach for the extraction of valuable characteristics from the AE signals for both ES. Each signal was first decimated and then bandpass filtered. The resulting signals were modelled using a TV-AR parametrical model for estimating a time-varying (TV) parametrical power spectral density (PSD) and TV poles. Finally an indicator based on tracking the pole of maximal energy was computed.

#### Signal pre-processing

The audio signal was first decimated to simplify the tracking of dynamical changes using the AR pole representation. The resulting signal was then bandpass filtered to focus the analysis in the frequency range significant for the used stethoscope.

#### Time-varying auto-regressive modelling for acoustic signature extraction

When an MID crosses a given tissue structure the resulting friction on the cutting edges produces an audio signal whose dynamics involve characteristics that are strongly variant in time. Additionally, when the MID passes through two different tissue structures (boundary between two different tissue layers for example) during and after the tissue transition the audio signal present important transient dynamics that can abruptly change. All these dynamical characteristics, together with the fact that the audio signals present significant background noise, and therefore a poor signal-to-noise-ratio (SNR), make the signal difficult to process and the information that this signal conveys must be decoded. What we propose in this work is to find an acoustic signature in the signal that provides information concerning tissue transition in the needle case and vessel perforation characteristic in the guide wire case.

Due to the signal characteristics described above, classical methods for stationary processes no longer can be used to follow the dynamical changes that this signal involves and cannot describe many conditions in processes where transient phenomena are involved. In the presence of time-varying characteristics the classical way to follow these variations is tracking and TV-AR modelling is well suited for extracting signature from audio signal using pole representation^22–24^. This acoustic signature was extracted in this work from the TV-AR spectrum and the dominant TV-AR pole.

The classical AR modelling for stationary processes is a well-known technique for parametrical spectral estimation and a huge amount of literature has been written showing its advantages over non-parametrical based methods (for detailed information about classical AR modelling we suggest to consult^25^). One advantage is that when an appropriate model is selected it presents a higher spectral resolution even in signals with poor SNR and using less data than classical methods. But another really important advantage for our work is that in its TV version it allows the decomposition of different TV dynamics through the pole representation allowing the tracking of those dynamical changes.

The main difference between the stationary AR version and the TV-AR one that we used in this work is that the parameters of the AR model are now time dependent, which results in a time-dependent representation of the transfer function:

1 $$H[z,n]=\frac{Y[z,n]}{E[z,n]}=\frac{1}{1+\sum _{k=1}^{p}{a}_{k}(n){z}^{-k}}$$

where *n* represent the sample time instant and *a*_*k*_(*n*) the time-variant AR parameters. This give rise to a time-varying spectrum

2 $${S}_{AR}(f,n)=\frac{1}{{|1+\sum _{k=1}^{p}{a}_{k}(n){e}^{j2\pi fn}|}^{2}}$$

As mentioned above dynamical changes of a nonstationary process can be tracked using a pole approach. Different poles should be associated with different dynamic components of the signal. Because of the complex properties of the MID/tissue interaction audio signal it would be very difficult to track each estimated pole. This is why we decided for this approach to track only one pole, the dominant one. We assume that transitions between tissue layers would be modelled mainly by one pole which at each time instant would contain the maximal energy of the spectrum. We can assume that when the MID passes from one medium to another, this dominant pole would change position and the time instant when the pole abruptly move would be the time instant of transition between two tissue layers.

For estimating the Time Varying Maximal Energy Pole (TV-MEP), first the poles *z*_*k*_(*n*) were obtained by finding the roots of the AR coefficient in the denominator of the time-varying pole representation transfer function that was obtained from equation (1)^22^:

3 $$H(z,n)=\frac{1}{\prod _{k=1}^{p}(1-{z}_{k}(n){z}^{-1})}.$$

Then the equation (3) was used for computing the evolution of the maximal energy pole, i.e., the pole that, at each time window, had the maximal spectral power. It was computed by calculating first the *r* time dependent resonant frequencies from the phase angle *θ*_*k*_(*n*) of the corresponding pole in the upper half of the complex plane as explained in^26^:

$${f}_{k}(n)=\frac{{\theta }_{k}(n)}{2\pi }={\tan }^{-1}(\frac{Im({z}_{k}(n))}{Re({z}_{k}(n))})\frac{{f}_{s}}{2\pi }$$

where *f*_*s*_ and ${f}_{k},k=1,2,\ldots ,r$ correspond to the sampling frequency and to the *r* resonant frequencies resulting from the poles, respectively. Then the spectral power *P*_*k*_ of the resonant frequency *k* was obtained from the real part of the residue term *r*_*k*_:

$$\begin{array}{rcl}{r}_{k}(n) & = & {z}^{-1}(z-{p}_{k}(n))H(z,n){|}_{z={z}_{k}(n)}\\ {P}_{k}(n) & = & 2{\sigma }^{2}Re({r}_{k}(n{))|}_{z={z}_{k}(n)}\end{array}$$

Finally at each time instant *n* the maximal energy pole was computed as the frequency belonging to the pole having the maximal power from the *r* resonant frequencies.

### Accession codes

The algorithms described in the Methods section are available to editors and referees upon request. For the needle ES an algorithm that has as input an audio signal and as output the detected time instants of the porcine tissue entry and exit can be provided. For the guide wire ES the full classification algorithm can be provided.

## Results and Discussions

In this section, qualitative and quantitative results are presented for both ES. We analyse performances on detecting abrupt dynamical changes produced by the needle tip during its entry and exit of tissue and on classifying a guide wire event as a perforation or as an artefact.

### Model parameter settings

In this work the TV-AR model parameters were computed over a sliding window of width *w* and an overlap of *Ov*. In each window, a *p* order AR model was used to estimate the AR parameters using the Yule-Walker method and for each of the windows the AR spectrum and poles were computed. The values of *w*, *Ov* and *p* were set to 110 ms, 50% and 30 respectively for the needle ES and 50 ms, 95% and 20 respectively for the guide wire ES.

For the needle ES, the bandpass filter consist of a 7*th* order Butterworth filter with a bandpass of 3–6 KHz. Due to the more transient characteristics of guide wire perforation dynamics the bandpass filter for this signal was implemented using Discrete Wavelet Transform (DWT). For that the signal was decomposed in 10 scales using a Daubechies DWT and finally reconstructed with selected middle-frequency wavelet scales as presented in^27^.

### Qualitative results

#### Biopsy needle tissue penetration analysis

The ES for the biopsy needle attempts to emulate different structures of tissue in order to analyse the different types of AE response that can be obtained as a result of the friction with cutting edges of the needle tip. The tested tissues were two fruits, persimmon and grape, and two chicken parts, breast and liver, all of them having different structure characteristics.

**Figure 4:**
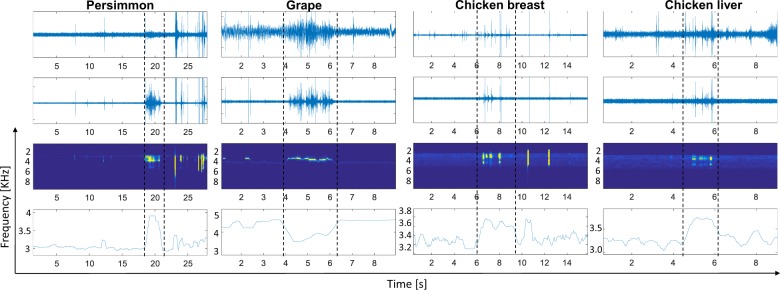
Four cases of tissue object penetration using fruits and chicken parts. From top to bottom the raw and filtered audio signals, the TVAR spectrum and the TV-MEP are displayed. The manually annotated time instants of tissue entry and exit are denoted by a dashed line.

Figure 4 displays the results of needle insertion in the four tested tissue objects. For each one the original signal, its bandpass filtered version, the TV-AR spectrum and the TV-MEP are shown. We can see in the original signal that only for the grape it is possible to clearly identify a dynamical change during the time interval between entry and exit from the tissue. However, it is difficult to determine the onset and offset of this dynamical change. In the other tissue object cases it is not at all evident when exactly the needle enters and passes through the object. Since the 3D printed box is not isolated from the outside the audio recordings involve different artefacts that can be more or less disturbing. It is therefore necessary to apply a signal processing strategy in order to enhance the information that otherwise stays hidden to the human eye. In this sense the bandpass filtered signals already enhances the information obtained from the penetration of the needle in the different tissues. The needle entering and leaving the persimmon becomes now visually evident and the TV-MEP significantly changes its frequency as a result of change of friction dynamics when the tip passes through the persimmon.

In chicken breast and liver, we can observe that the friction dynamics during needle penetration are more transient than in the fruits, resulting in a more difficult visualization of the process even after the filter is applied. AR modelling, as parametrical technique to analyse data, can help through the pole representation to extract more valuable and analytical information from friction dynamics, as can be seen in the behaviour of the TV-MEP.

**Figure 5:**
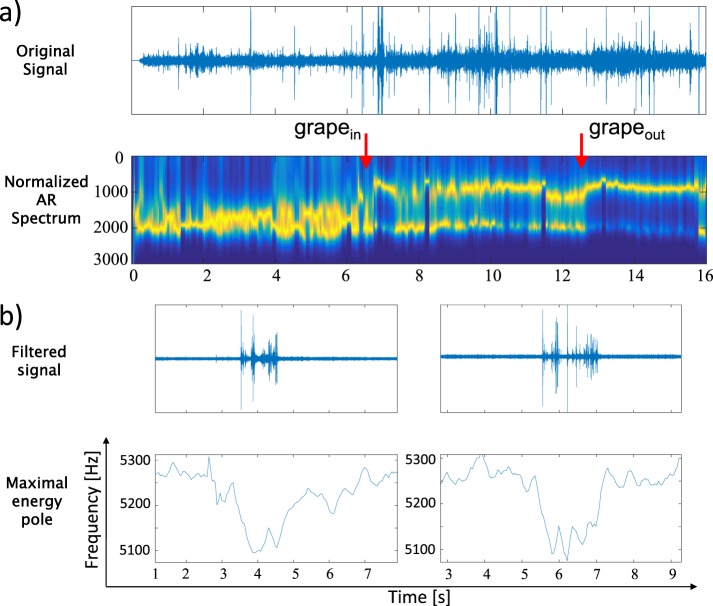
(**a**) Normalised TV-AR spectrum of a grape. (**b**) AE recordings of needle penetration in grape at two different depths and their respective TV-MEP.

The analysis of the grape case shows interesting results starting with the normalized TV-AR spectrum that is shown in Fig. 5a. The grape is a fruit with internal inhomogeneities as a combination of seeds and water and with that its TV-AR spectrum is quite illustrative. We can observe that before and after the entry and exit from the grape the main frequencies are more stable and concentrated in a narrowband location. This can be explained by the homogeneity of the gelatine. When the needle is inside the grape the frequency band is wider, certainly due to the unstable beating of the needle between seeds and water.

Another important analysis can be observed in Fig. 5b. In this figure AE recordings of grapes located at two different depths, 5 and 10 cm, are displayed. We can see in the filtered audio signal that there is nearly no difference in intensity between both grapes and the TV-MEP for both cases are similar. This would suggest that the audio is propagated without significant losses from both locations.

Finally it is important to look at the difference in Fig. 2 between the needle entry and exit dynamics in the audio and force signals. It is possible to observe that the transition from gelatine to fat, when the needle enter to the tissue, is abrupt in the audio while in the force is slow. This would suggest that the transition in the process between two states (needle passing through two different tissues) could be abruptly detected in the audio signal but not in the force signal. Furthermore, the exit from the fat is evident in the audio signal but not in the force signal, indicating that the dynamical changes occurring in the process when a needle passes a layer boundary are not lost in the audio signal compared with the force signal when the event occurs deeper in the body.

#### Guide wire perforation audio signal characteristics

**Figure 6:**
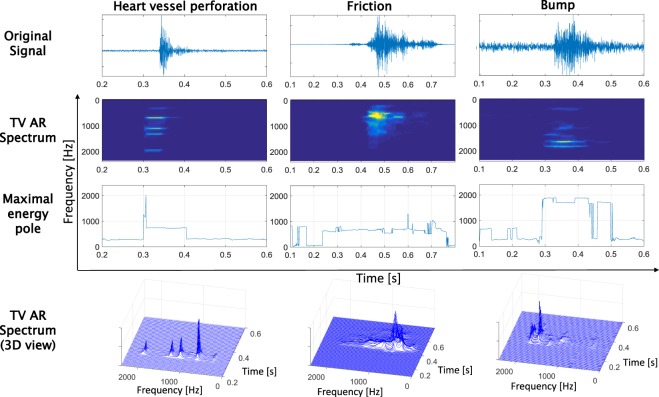
Guide wire audio signals with the respective TV-AR spectrums and maximal energy pole signals for three real cases (adapted from^21^).

Figure 6 shows three real audio signals obtained from the proximal end of the guide wire. Three different guide wire event cases are depicted to describe the differentiating signal patterns: heart vessel perforation, friction and a guide wire bump. For each case, the original signal, the TV-AR spectrum and the TV-MEP are shown. Additionally, we display a 3D option for the visualization of the spectrum. The 2D display of the spectrum allows to see the time spectral lines and the 3D can help us to have a better insight on the frequency power distribution over time. A visual analysis of the time-varying patterns of the spectrum and of the TV-MEP allows to verify the following:

- The TV-AR spectrum shows main frequency components that are stable in time during a perforation (inside the segment of higher energy in the TV spectrum), unlike an artefact where the frequencies are more disperse in time. That is equivalent to say that it is possible to observe a higher spectral disorder when an artefact occurs than when a perforation occurs.
- During a perforation some characteristic segments can be distinguished in the TV-MEP: 1) a strong overshoot represented by a fast and short rise of the pole frequency, 2) a plateau just after the overshoot until the signal goes back to a stationary stage. It is possible to observe that the overshoot is not present when an artefact occurs and also that the plateau is much more stable when a perforation occurs than when an artefact is generated.

This analysis leads us to believe that a perforation can be identified from other occurring events, since it leaves a clear audio signal trace in the time-frequency domain. First, the overshoot is an important characteristic of a perforation indicating a high concentration and release of energy just before and just after the guide wire tip crosses the vessel wall. We can assume that different elasticities of vessel structures could result in different patterns of the overshoot. But this overshoot should be present when a perforation occurs. Secondly, the audio signal dynamics in the TV-MEP just after the overshoot are stable, which can be explained by the fact that after exiting the vessel wall the dynamic comes back to a stationary state through a damped stable frequency that should be a transformation of the natural frequency of the tissue. This dominant frequency has high spectral energy and is always characterized by a unique pole. The purity of the main frequencies of the spectrum in terms of their stability over time, which is clearly visible in both 2D/3D visualization of the spectrum, confirms the clear response of the vessel wall after excitation.

### Quantitative results

#### Needle experimental setup

For each recording belonging to the needle database, the entry and exit time instants were automatically detected using a simple CUSUM hypothesis testing over the obtained TV-MEP signal^28^. These time instants were compared with the manual references by computing the absolute values of the time errors between automatic and manual annotations.

**Figure 7:**
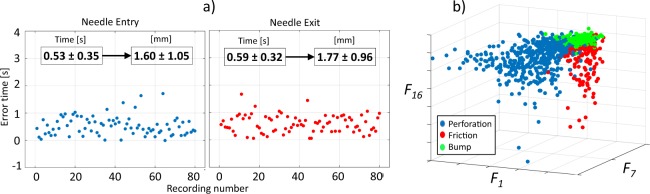
Summary of quantitative results obtained by the approach: (**a**) results of entry and exit detection of the needle for the 80 audio recordings, (**b**) example of three guide wire features for the 875 audio recordings separated in heart vessel perforation (blue), friction (red) and guide wire bump (green).

In Fig. 7a the errors between manual and automatic annotations are shown for each one of the 80 recordings for both, needle entry and exit from the *ex-vivo* porcine tissue. In all the cases the TV-MEP was sensible to needle entry and exit. The average and standard deviation of the detection errors was 0.53 ± 0.35 seconds for needle entry and 0.59 ± 0.32 seconds for needle exit. If we consider that the automatic needle insertion velocity with the testing machine was 3 mm/s the error in millimetres is equivalent to 1.60 ± 1.05 mm and 1.77 ± 0.96 mm respectively for entry and exit.

For the recordings performed at the additional velocities of 5 and 8 mm/s the average and standard deviation of the detection errors was 0.11 ± 0.29 and 0.16 ± 0.30 seconds for needle entry and −0.09 ± 0.51 and 0.002 ± 0.40 seconds for needle exit, respectively. Considering the velocities the errors are equivalent to 0.56 ± 1.44 mm entry and −0.43 ± 2.53 mm exit for the velocity insertion of 5 mm/s and 1.27 ± 2.37 mm entry and 0.02 ± 3.23 mm exit for the velocity insertion of 8 mm/s.

It is possible to observe that in terms of detection errors in seconds the results between the three tested velocities are similar for needle entry and exit. However in terms of distance we can observe that the standard deviation increases directly with the velocity. This is explained by the fact that an error has a major effect when the velocity is higher.

#### Guide wire experimental setup

For the guide wire 16 features were extracted from the TV-MEP and from the TV-AR spectrum for each one of the 875 recordings from the database. These features are computed following the qualitative analysis made previously about the dynamical characteristics of a perforation. For the explanation of the extracted features let *P*_*m*_(*n*) be the TV-MEP signal at time instant *n* and *S*_*AR*_(*f*, *n*) be the TV-AR spectrum computed from equation (2) at time instant *n* and frequency *f*. Let also *t*_*on*_ and *t*_*off*_ be the time instant of beginning and end of the occurring event (perforation or artefact), which are computed following the method presented in^29^. The proposed features can be divided in three groups: overshoot based features, plateau based features and TV-AR spectrum based features.

Overshoot based features: The main objective of this type of features is to provide quantitative information concerning the overshoot of *P*_*m*_(*n*) that is usually present in guide wire perforations. We compute 6 overshoot based features as:

$${F}_{1}=\frac{O{S}_{H}}{O{S}_{W}}\cdot \frac{\sum _{n=O{S}_{on}}^{n=O{S}_{off}}{P}_{m}(n)}{\sum _{n={t}_{on}}^{n={t}_{off}}{P}_{m}(n)}$$

$${F}_{2}=\mathop{{\rm{\max }}}\limits_{n}[{P}_{m}(n)]{|}_{{t}_{on}}^{{t}_{off}}$$

$${F}_{3}=\mathop{{\rm{\max }}}\limits_{n}[{P}_{m}(n){\overline{S}}_{f}(n)]{|}_{{t}_{on}}^{{t}_{off}}$$

$${F}_{4}=\frac{{F}_{2}}{\sum _{n={t}_{on}}^{n={t}_{off}}{P}_{m}(n)}$$

$${F}_{5}=\frac{{F}_{3}}{\sum _{n={t}_{on}}^{n={t}_{off}}{P}_{m}(n){\overline{S}}_{f}(n)}$$

$${F}_{6}=\frac{{F}_{3}}{\frac{1}{{t}_{off}-{t}_{on}}\sum _{n={t}_{on}}^{n={t}_{off}}{P}_{m}(n){\overline{S}}_{f}(n)}$$

where *OS*_*H*_, *OS*_*W*_, *OS*_*on*_ and *OS*_*off*_ are respectively the height, width, starting time and ending time of the overshoot and ${\overline{S}}_{f}(n)$ is the frequency-averaged TV-AR spectrum computed as:

$${\overline{S}}_{f}(n)=\frac{1}{{N}_{f}}\sum _{f=0}^{f={f}_{s}\mathrm{/2}}{S}_{AR}(n,f)$$

where *N*_*f*_ is the number of samples in frequency of the TV-AR spectrum and *f*_*s*_ correspond to the sampling frequency.

Plateau based features: These features are based on the stability of the plateau characteristics in *P*_*m*_(*n*). We consider a plateau in *P*_*m*_(*n*) as an event occurring just after the overshoot, which involves low signal fluctuations. Therefore the plateau is estimated using the derivative of *P*_*m*_(*n*). If this derivative is continuously low enough then the interval is considered as a plateau. Following this concept three plateau signal intervals are computed *PL*_1_(*n*), *PL*_2_(*n*) and *PL*_3_(*n*) by using three different threshold values to the fluctuations in the derivative of *P*_*m*_(*n*). We compute 8 plateau based features as:

$${F}_{7}=\frac{Var[P{L}_{1}(n)]}{Length[P{L}_{1}(n)]}$$

$${F}_{8}=Peaks[\frac{d{P}_{m}(n)}{dn}]{|}_{{t}_{on}}^{{t}_{off}}$$

$${F}_{9}=Peaks[\frac{d{P}_{m}(n)}{dn}]{|}_{{t}_{on}}^{{t}_{on}+{\rm{\Delta }}}$$

$${F}_{10}=\frac{Length[P{L}_{1}(n)]}{{t}_{off}-{t}_{on}}$$

$${F}_{11}=\frac{Length[P{L}_{1}(n)]}{Std(P{L}_{1}(n))}$$

$${F}_{12}=\frac{Length[P{L}_{2}(n)]}{{t}_{off}-{t}_{on}}$$

$${F}_{13}=Length[P{L}_{2}(n)]$$

$${F}_{14}=Length[P{L}_{3}(n)]$$

where Δ is an interval of time starting just after the onset of the plateau (maximal energy time interval of the event). The functions *Length*, *Std* and *Var* correspond respectively to the length, standard deviation and variance of the plateau. The function *Peaks* compute the number of peaks present in the derivative of *P*_*m*_(*n*).

TV-AR Spectrum based features: These features have as main objective to quantify the stability of the main time-varying frequency components. For that two simple correlation features are used over the TV-AR spectrum, one involving the complete event and other one involving only the time interval of maximal energy of the event:

$${F}_{15}=Mean\{Corr[{S}_{AR}(f,n)]\}{|}_{{t}_{on}}^{{t}_{off}}$$

$${F}_{16}=Mean\{Corr[{S}_{AR}(f,n)]\}{|}_{{t}_{on}}^{{t}_{on}+{\rm{\Delta }}}$$

where *Corr* is the correlation of the matrix and *Mean* is the average of the correlations.

Figure 7b shows a 3D scatter plot for the values of the 875 guide wire audio signal obtained for three different features belonging each one to a different type of feature: the overshoot based *F*_1_, the plateau based *F*_7_ and the TV-AR Spectrum based *F*_16_. This scatter shows different location for the points belonging to heart vessel perforation compared with the artefact events.

In order to classify between heart vessel perforation and artefact a Support Vector Machine (SVM) was used as the tool for binary classification (perforation or artefact). SVMs are widely used in applications such as bioinformatics, text mining, face recognition and image processing. They are based on supervised learning and margin maximization, meaning that a labelled dataset (perforations labelled with 1 as target value, artefacts with 0 as target value) is fed to the algorithm to create a high dimensional hyperplane, which serves as the desired decision boundary with maximal distance between the classes^30^. In this work a 16-dimensional space is used.

A balanced training data set plays a crucial role in the performance of SVM^31^. In order to obtain a meaningful result, the same proportion of perforations and artefacts were used for training. Thus, 100 recorded perforations were used along with 50 frictions and 50 bump recordings for training. For the subsequent test of the trained SVM 460 heart vessel perforation recordings, 115 friction recordings and 100 bump recordings were used.

Table 1 shows the obtained results of the SVM classification where we can see that from 460 tested heart vessel perforations the algorithm recognize correctly 418 recordings. It is possible to see also that from the 215 tested artefact only 10 of them were assigned as perforation. This demonstrates that the assumptions on overshoot, plateau, and time-varying spectrum were highly useful for distinguishing between a perforation and an artefact.

**Table 1:** Results of classification between heart vessel perforation and artefact using SVM classification.

True positive (correctly recognized heart perforation)	418
False negative (wrongly assigned heart perforation)	42
False positive (wrongly assigned artefact)	10
True negative (correctly recognized artefact)	205
Sensitivity	90.9%
Specificity	95.3%

## Conclusions

The presented research approach shows a novel and relatively simple method to obtain valuable information for MID guidance and tissue/device interactions using an attached microphone that records propagated sound over the devices shaft for further analysis and evaluation. These interactions starting at the tip of an interventional device can be picked up and detected on the proximal end of a standard clinically used device. Even if this work present results from experiments performed in a laboratory controlled environment, where many sources of physiological and not physiological artefacts are not present, we strongly believe that due to the clear dynamical changes in the signal, performances of the presented approach should not be significantly affected in real clinical conditions.

The experiments with an implemented database for both MIDs show that valuable information can be extracted from the audio signal through the TV-MEP. It is sensitive to tissue boundary transitions and has a distinguishable signature when the guide wire perforates a vessel. The audio induced by the friction on the cutting edges in needle insertion and perforation of a guide wire are events that involve signal dynamics containing specific time-variant frequency components and dominating energies. For this reason it should be possible to distinguish these events, clinically important, from events or artefacts induced by other unwanted sources during the process.

The results indicate that a first use of this approach should be for verification and/or visual haptic feedback for real time tissue/tissue passage. The obtained processed audio signal contains additional complementary feedback information to the one provided by image guidance. For example, if integrated with ultrasound this would allow verification of needle tip position even when the tip is difficult to distinguish, which happens quite frequently in applications such as regional anaesthesia, where the angle between the US probe and the needle is too small. Another application could be for surgical robot guidance where the audio information could correct or verify a position or be used for haptic robotic feedback.

For applications with relatively clear boundary structure, the presented approach will help to obtain clearly distinguishable and automatically detectable dynamical changes when the MID passes through tissue structures.

We were able to clearly identify boundaries and we can observe that each tested tissue leaves a different acoustic signature when analysed in a time-varying parametrical context. A next development step will be to study whether it is possible to classify tissue through this approach. For that, advanced signal processing algorithms should be developed for taking into account the non-linearities, inhomogeneities and elasticity characteristics of a given tissue.

Also needed is a methodology for translating decoded information into a friendly graphical user interface or acoustic signal and to study and evaluate human performances with respect to a real clinical intervention.

## Electronic supplementary material

Needle concept

Supplementary information

## Data Availability

The datasets involving the database for needle insertion and guide wire perforation generated and analysed during the current study are available in the *figshare* repository 10.6084/m9.figshare.c.4035041.

## References

[1] Abolhassani N, Patel R, Moallem M (2007). Needle insertion into soft tissue: A survey. Medical engineering & physics.

[2] van Gerwen DJ, Dankelman J, van den Dobbelsteen JJ (2012). Needle–tissue interaction forces–a survey of experimental data. Medical engineering & physics.

[3] Robert AL, Chagnon G, Bricault I, Cinquin P, Moreau-Gaudry A (2013). A generic three-dimensional static force distribution basis for a medical needle inserted into soft tissue. Journal of the mechanical behavior of biomedical materials.

[4] Fukushima Y, Naemura K (2014). Estimation of the friction force during the needle insertion using the disturbance observer and the recursive least square. ROBOMECH Journal.

[5] Trejos A, Patel R, Naish M (2010). Force sensing and its application in minimally invasive surgery and therapy: a survey. Proceedings of the Institution of Mechanical Engineers, Part C: Journal of Mechanical Engineering Science.

[6] Bao X, Li W, Lu M, Zhou Z (2016). Experiment study on puncture force between mis suture needle and soft tissue. Biosurface and Biotribology.

[7] Kern M (2016). Comparing ffr tools: New wires and a pressure microcatheter. Cathet Lab Digest.

[8] Tesei, M. *et al*. A cost-effective, non-invasive system for pressure monitoring during epidural needle insertion: Design, development and bench tests. In *Engineering in Medicine and Biology Society (EMBC), 2016 IEEE 38th Annual International Conference of the*, 194–197 (IEEE, 2016). 10.1109/EMBC.2016.7590673 28268312

[9] Kalvøy H (2009). Impedance-based tissue discrimination for needle guidance. Physiological measurement.

[10] Park, J. & Park, I. Development of multi-spot impedance sensing biopsy needle based on attachable and flexible sensor film. In *Engineering in Medicine and Biology Society (EMBC), 2016 IEEE 38th Annual International Conference of the*, 4788–4791 (IEEE, 2016). 10.1109/EMBC.2016.7591798 28269341

[11] Okamura A (2005). Detection of embolic particles with the doppler guide wire during coronary intervention in patients with acute myocardial infarction: efficacy of distal protection device. Journal of the American College of Cardiology.

[12] Ferrari M, Werner GS, Bahrmann P, Richartz BM, Figulla HR (2006). Turbulent flow as a cause for underestimating coronary flow reserve measured by doppler guide wire. Cardiovascular ultrasound.

[13] Matinfar, S. *et al*. Surgical soundtracks: Towards automatic musical augmentation of surgical procedures. In *International Conference on Medical Image Computing and Computer-Assisted Intervention*, 673–681 (Springer, 2017).

[14] Black, D., Hansen, C., Nabavi, A., Kikinis, R. & Hahn, H. A survey of auditory display in image-guided interventions. *International Journal of Computer Assisted Radiology and Surgery* 1–12 (2017). 10.1007/s11548-017-1547-z PMC5591070 28275890

[15] Lauro C, Brandão L, Baldo D, Reis R, Davim J (2014). Monitoring and processing signal applied in machining processes–a review. Measurement.

[16] Aggelis D, Kordatos E, Matikas T (2011). Acoustic emission for fatigue damage characterization in metal plates. Mechanics Research Communications.

[17] Kapur RA (2016). Acoustic emission in orthopaedics: A state of the art review. Journal of biomechanics.

[18] Pohl, B. M., Jungmann, J. O., Christ, O. & Hofmann, U. G. Automated drill-stop by svm classified audible signals. In *Engineering in Medicine and Biology Society (EMBC), 2012 Annual International Conference of the IEEE*, 956–959 (IEEE, 2012). 10.1109/EMBC.2012.6346091 23366052

[19] Jin, H. *et al*. Intraoperative control for robotic spinal surgical system with audio and torque sensing. In *Multisensor Fusion and Information Integration for Intelligent Systems (MFI), 2014 International Conference on*, 1–6 (IEEE, 2014).

[20] Sun, Y., Jin, H., Hu, Y., Zhang, P. & Zhang, J. State recognition of bone drilling with audio signal in robotic orthopedics surgery system. In *Intelligent Robots and Systems (IROS 2014), 2014 IEEE/RSJ International Conference on*, 3503–3508 (IEEE, 2014).

[21] Illanes, A., Schaufler, A., Maldonado, I., Boese, A. & Friebe, M. Time-varying acoustic emission characterization for guidewire coronary artery perforation identification. In *Computing in Cardiology Conference (CinC), 2017* (2017).

[22] Mainardi LT, Bianchi AM, Baselli G, Cerutti S (1995). Pole-tracking algorithms for the extraction of time-variant heart rate variability spectral parameters. IEEE Transactions on Biomedical Engineering.

[23] Eom KB (1999). Analysis of acoustic signatures from moving vehicles using time-varying autoregressive models. Multidimensional Systems and Signal Processing.

[24] Scalassara PR (2007). Autoregressive decomposition and pole tracking applied to vocal fold nodule signals. Pattern recognition letters.

[25] Manolakis, D. G., Ingle, V. K. & Kogon, S. M. *Statistical and adaptive signal processing: spectral estimation, signal modeling, adaptive filtering, and array processing*. (McGraw-Hill Boston, 2000).

[26] Thanagasundram S, Spurgeon S, Schlindwein FS (2008). A fault detection tool using analysis from an autoregressive model pole trajectory. Journal of Sound and Vibration.

[27] Illanes, A. & Haritopoulos, M. Fetal heart rate feature extraction from cardiotocographic recordings through autoregressive model’s power spectral-and pole-based analysis. In *Engineering in Medicine and Biology Society (EMBC), 2015 37th Annual International Conference of the IEEE*, 5842–5845 (IEEE, 2015). 10.1109/EMBC.2015.7319720 26737620

[28] Basseville, M. *et al*. *Detection of abrupt changes: theory and application*, vol. 104 (Prentice Hall Englewood Cliffs, 1993).

[29] Meenakshi GN, Ghosh PK (2015). Robust whisper activity detection using long-term log energy variation of sub-band signal. IEEE Signal Processing Letters.

[30] Kecman, V. Support vector machines–an introduction. In *Support vector machines: theory and applications*, 1–47 (Springer, 2005).

[31] Akbani, R., Kwek, S. & Japkowicz, N. Applying support vector machines to imbalanced datasets. In *European conference on machine learning*, 39–50 (Springer, 2004).

