# Supplementary material for: Novel clinical device tracking and tissue event characterization using proximally placed audio signal acquisition and processing
Source: Sci Rep. 2018 Aug 13;8:12070. doi: 10.1038/s41598-018-30641-0 (PMC6089924; doi:10.1038/s41598-018-30641-0)
Supplement: Supplementary file 2 — Supplementary information [file 41598_2018_30641_MOESM2_ESM.pdf]

SUPPLEMENTARY INFORMATION: Novel clinical device tracking and tissue event characterization using proximally placed audio signal acquisition and processing

Alfredo Illanes*[1], Axel Boese[1], Iván Maldonado[1], Ali Pashazadeh[1], Anna Schaufler[1], Nassir Navab[2], and Michael Friebe[1]

[1]INKA Intelligente Katheter, Otto-von-Guericke-Universität Magdeburg
[2]Fakultät für Informatik, Technische Universität München

*alfredo.illanes@ovgu.de

Supplementary note 1: Overshoot parameters computation

This supplementary note has as main objective to explain how the overshoot parameters (height OS_H, width OS_W, starting time OS_{on} and ending time OS_{off}) were extracted from the TV-MEP $P_m(n)$ for computing the feature F_1 (see Section 3.3.2 in the main text). As shown in Figure **??** the derivative of $P_m(n)$ is first computed and then the time instant where the maximal positive (t_{max}) and minimal negative (t_{min}) peaks of the derivative occurs are automatically located. The time instant of the peak of the overshoot (t_{peak}) is computed then as the maximal value in $P_m(n)$ between t_{max} and t_{min}. The starting time of the overshoot was set at the same time instant of maximal positive derivative. The ending point of the overshoot was set at the time instant of the derivative zero-crossing point occurring just after the minimal negative peak of the derivative. In summary the overshoot parameters are computed as:

$$
\begin{aligned}
OS_{on} &= t_{max} \\
OS_{off} &= ZeroCrossing[dP_m(n)/dn] \\
OS_H &= P_m(t_{peak}) - P_m(OS_{on}) \\
OS_W &= OS_{off} - OS_{on}
\end{aligned}
$$

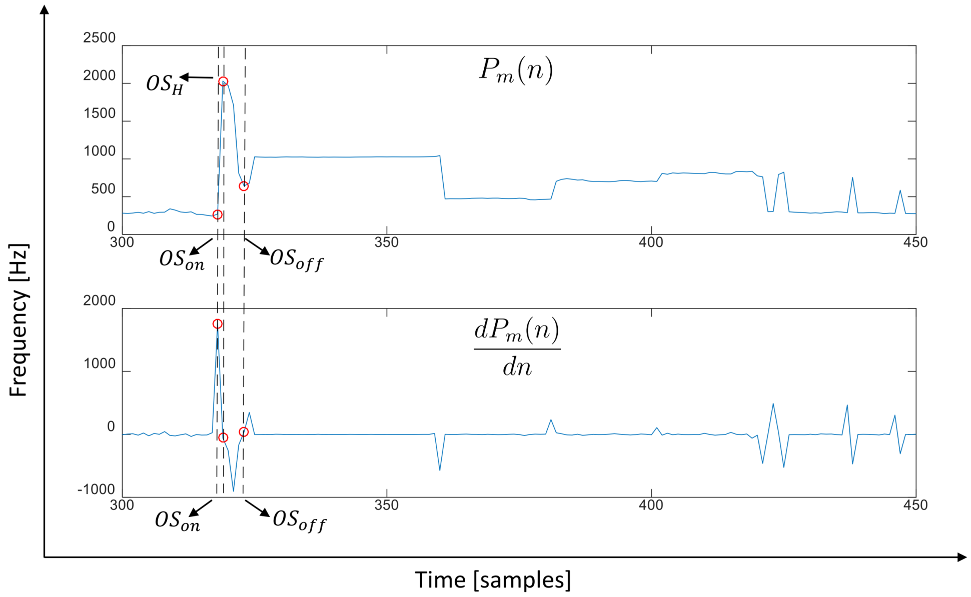

Figure S1. TV-MEP and its derivative for computation of the main parameters of an overshoot.

Supplementary note 2: Computation of the three versions of the plateau signal

In this supplementary note the extraction of the three plateau signals ($PL_1(n)$, $PL_2(n)$ and $PL_3(n)$) used for computer the plateau-based features is explained. As presented in Section 3.3.2 of the main text, a plateau in $P_m(n)$ is an event occurring just after the overshoot involving low fluctuations. We compute three plateau signals from $P_m(n)$ that have as only difference the threshold use for classifying a segment as a low fluctuation one. First the derivative of $P_m(n)$ is computed and when this derivative is less or equal than a selected threshold λ we consider a segment in $P_m(n)$ as stable. At the top of Figure **??** we can see the computation of the stable segments in $P_m(n)$ using three different λ values (λ_1, λ_2 and λ_3). Each one of the three plateau correspond to the longest stable segment for each λ value.

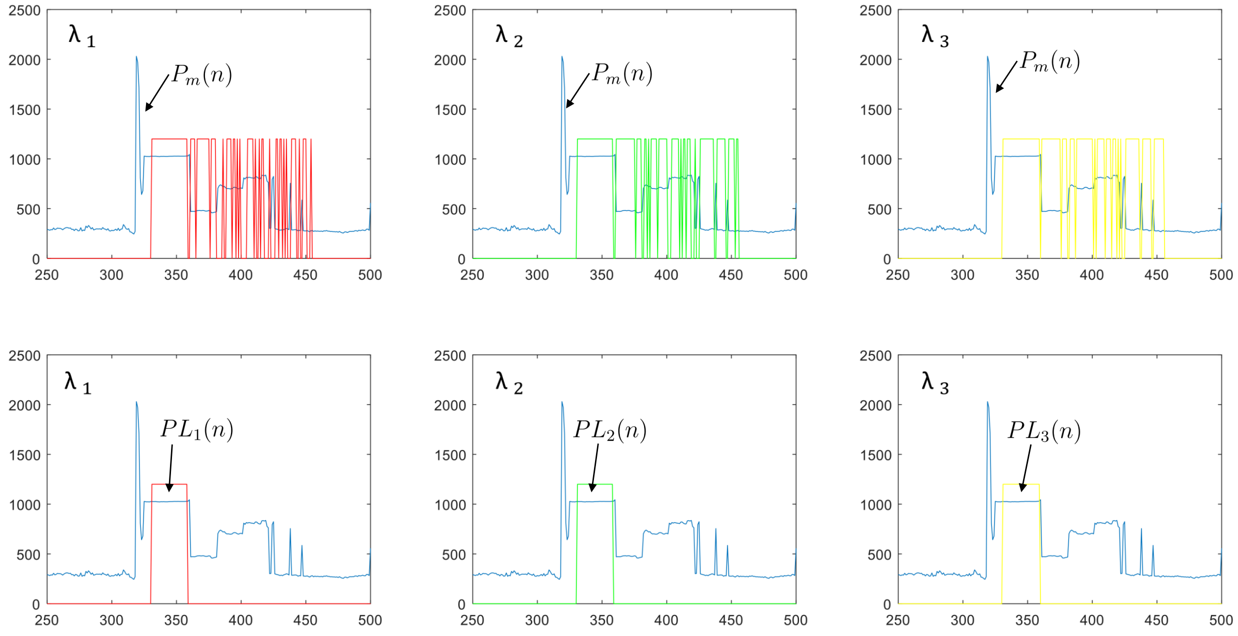

Figure S2. Computation of the three plateau signals from the TV-MEP using three different fluctuation threshold.

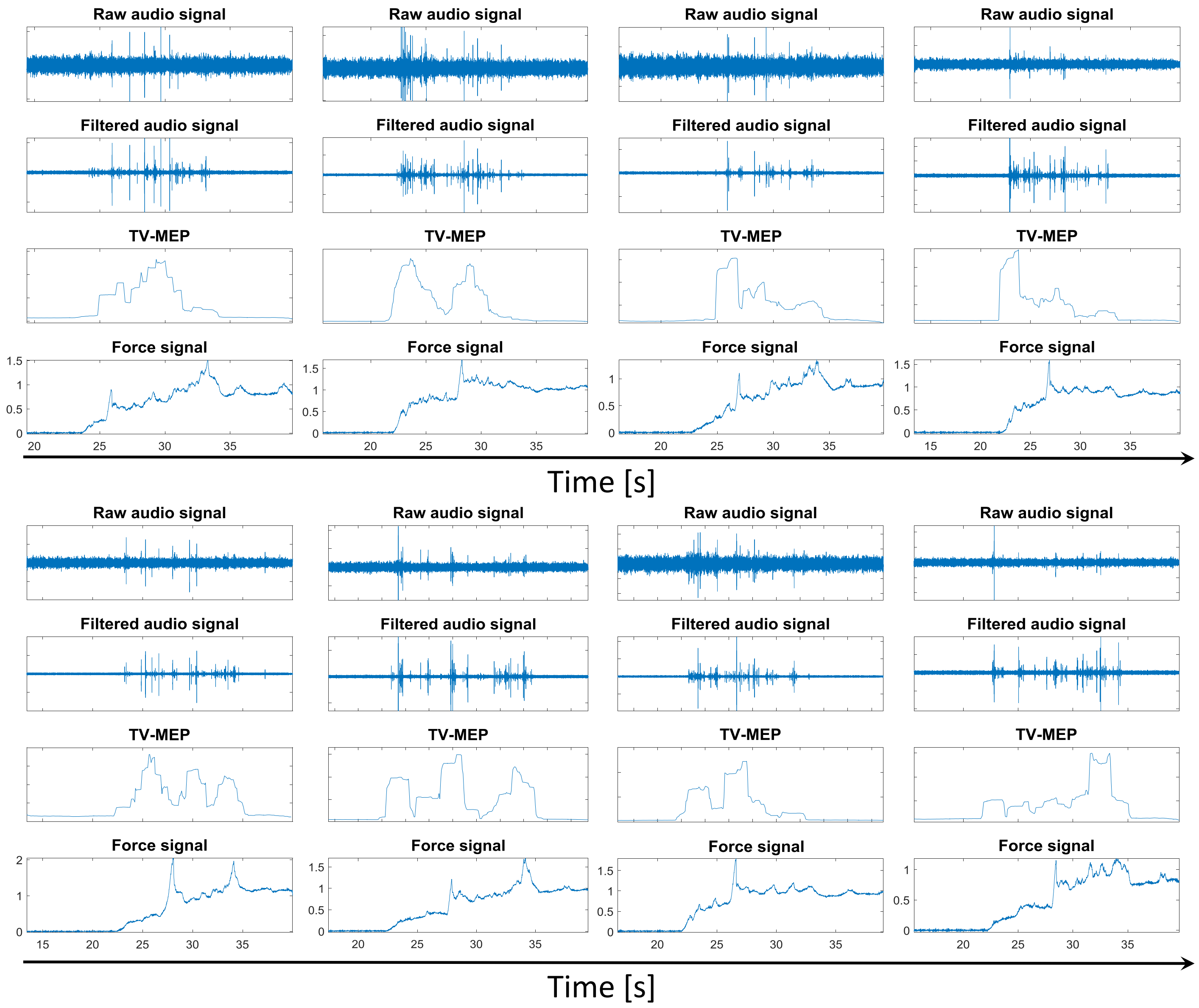

Figure S3. Eight examples from the needle insertion in porcine tissue database. In the top we can see the raw audio recording, then the pre-processed audio and the Time-varying Maximal Energy Pole are displayed and finally in the bottom the recorded force during needle insertion is shown.

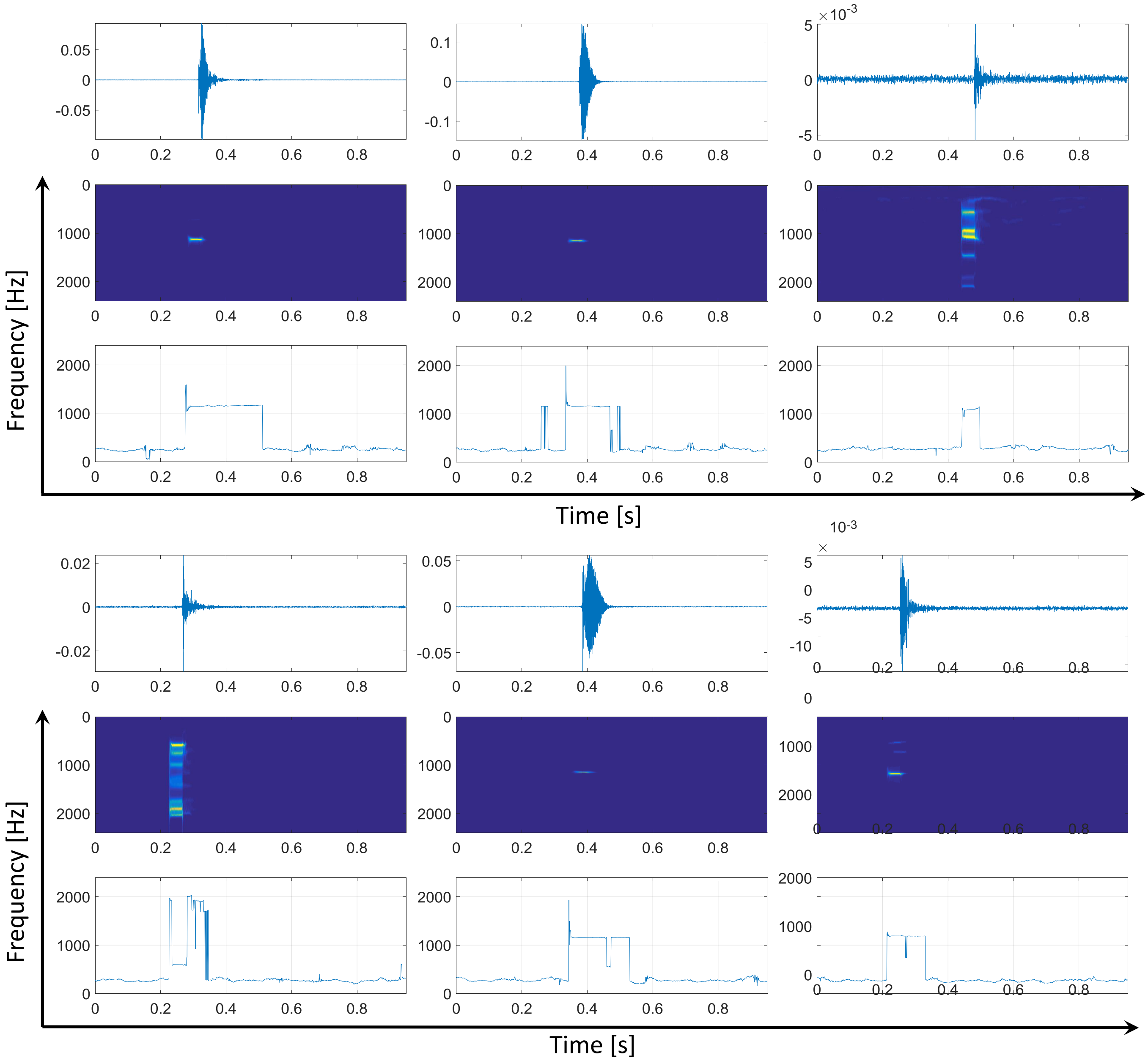

Figure S4. Six examples of results for guide wire heart perforation. In the top we can see the raw audio recording, in the middle the Time-varying auto-regressive spectrum and in the bottom the Time-varying Maximal Energy Pole.

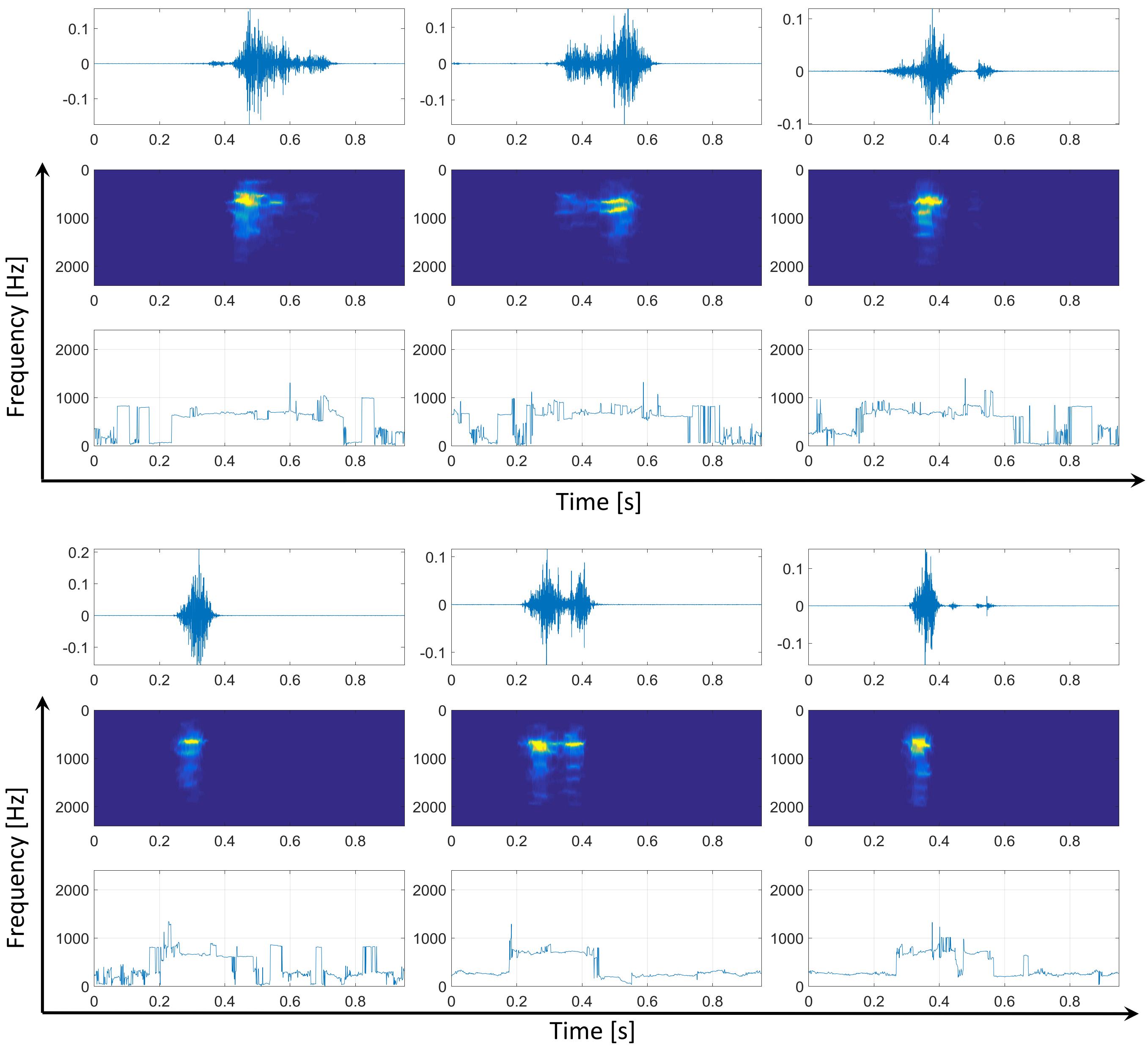

Figure S5. Six examples of results for friction between guide wire and the wall vessel. In the top we can see the raw audio recording, in the middle the Time-varying auto-regressive spectrum and in the bottom the Time-varying Maximal Energy Pole.

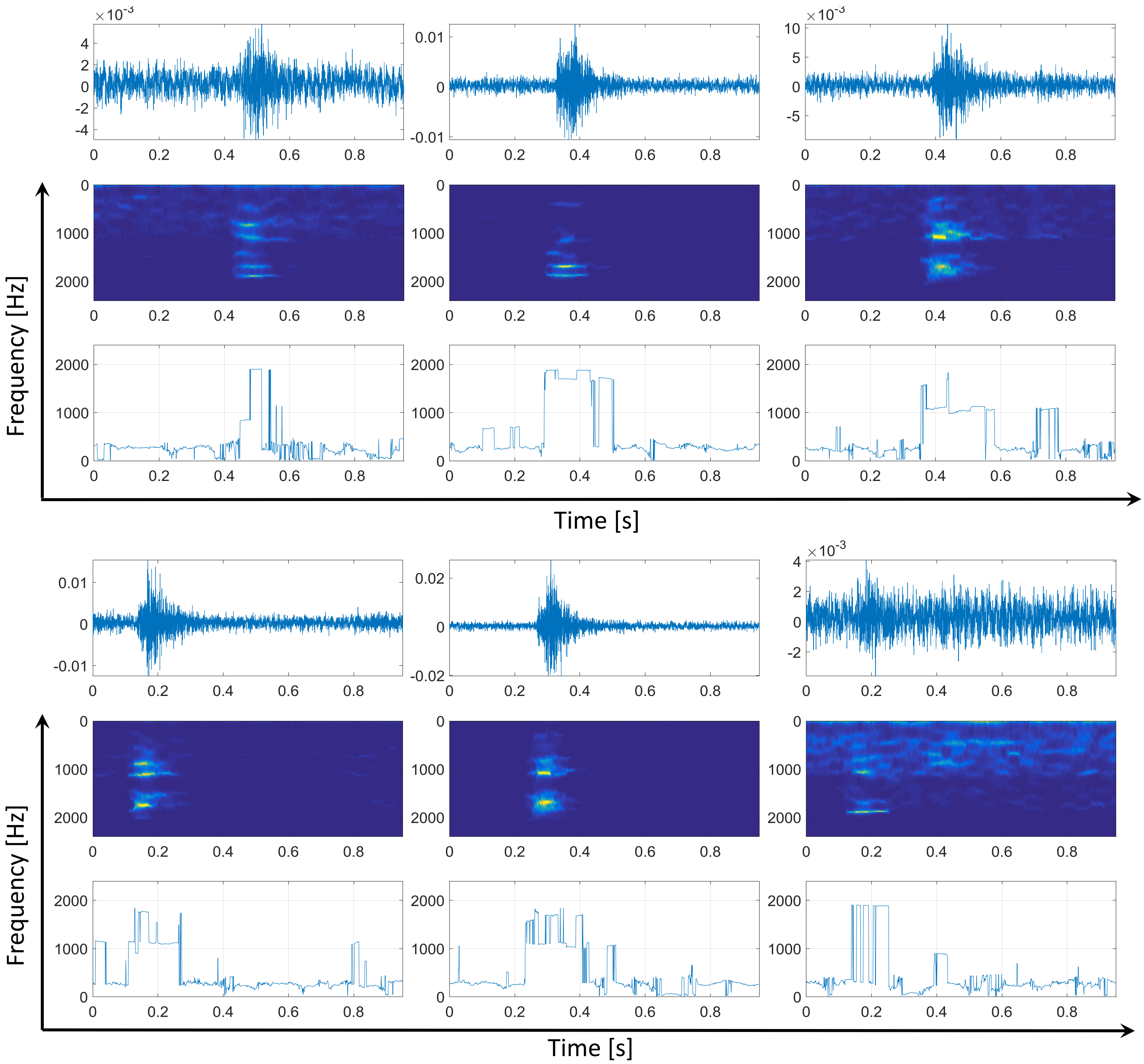

Figure S6. Six examples of results for guide wire bump. In the top we can see the raw audio recording, in the middle the Time-varying auto-regressive spectrum and in the bottom the Time-varying Maximal Energy Pole.
